# Editorial: Insights in occupational health and safety: 2021

**DOI:** 10.3389/fpubh.2022.975534

**Published:** 2022-07-29

**Authors:** Luigi Vimercati

**Affiliations:** Section of Occupational Medicine, Interdisciplinary Department of Medicine, University of Bari, Bari, Italy

**Keywords:** occupational health, occupational safety, prevention, worker, workplace

Occupational Medicine has always been concerned with preventing health effects caused by working conditions, and with promoting and maintaining the highest level of physical, mental, and social wellbeing of workers in all occupations. This “*Insights in Occupational Health and Safety: 2021*” Research Topic, of 13 articles, reflects the geographic variety of occupational health and safety researchers with contributors from Asia, Europe, America, Africa, and Oceania. It includes forward looking contributions focused on new occupational risk factors, current challenges, recent advances and future perspectives in the field of Occupational Health and Safety. From this perspective, there has been an increasing effort in recent years to investigate the causes of occupational musculoskeletal disorders and to take action to prevent them. Regarding this issue Lourenço and Luís investigated how musculoskeletal disorders in welders may influence their quality of life, finding a higher incidence of symptoms in welders in comparison to non-welders. Furthermore, the presence of musculoskeletal disorders, particularly in the lumbar area, was related to an increased bodily pain and decreased health-related quality of life. Aleku et al. found that the frequency rate of low back pain was high amongst healthcare workers (HCWs). In this occupational category, this problem could be related to workloads due to the COVID-19 pandemic. A high workload, in fact, not only adversely affects safety, but also negatively affects job satisfaction and, as a result, contributes to high turnover and staff shortages. Li, Hu, Liu, et al. performed a physician comprehensive workload analysis among Chinese physicians. The assessment of workload proposed by the Authors could be a potential application for hospital managers to further determine and accurately identify physicians with high workload. Saleem et al. pointed out that it is appropriate to intervene on safety of some groups of workers such as those working in construction. Despite significant efforts over the past several decades, occupational safety is still, in fact, a highly relevant and serious issue worthy of academic attention (Liu L. et al.). On this basis, the challenge for occupational medicine, in the near future, is to increase occupational safety and prioritize workplace health promotion and job satisfaction (Liu D. et al.). Only through this process it would be possible to face the new challenges related to health effects of gig-economy. The gig economy is a rising phenomenon globally, where gig workers present “alternative work arrangements” for pieces of jobs (“gigs”) or more generally short-term contract, which are mainly agreed upon *via* digital platforms for different services, including food delivery or transportation. Focusing on the health effects among gig workers is of great public health relevance and epidemiological studies could better stimulate policies to design and implement preventive health measures (Freni-Sterrantino and Salerno).

Moreover, studies on interactions between environmental and genetic factors are assuming more and more urgency and importance. In this context the third follow-up of the study on occupational allergy risks cohort, in Germany, offers the opportunity to analyze the course of asthma and allergies and their associations to environmental, occupational, and psychosocial risk factors over more than 20 years from childhood to adulthood (Forster et al.). Fu et al. investigated acute respiratory distress syndrome caused by occupational exposure to waterproofing spray, while Hall et al. recommend ongoing research to understand links between military exposures and veteran health.

Finally, the coronavirus disease 2019 (COVID-19) pandemic has also changed the public opinion on the need for safe practices in the workplace and has focused the attention of governments and institutions worldwide on the fundamental role that the occupational safety and health services play. In particular healthcare workers (HCWs) are a group at high risk of infection in general and specifically of SARS-CoV-2 infection (Keleb et al.), even with psychological harm (Li, Hu, Chen et al.). Since the start of the pandemic several studies have been carried out on prevalence of COVID-19, and on related risk factors in this group of workers. An Italian multicenter study, conducted during the first months of the pandemic, revealed that the prevalence of infection in HCWs varied across centers, with results collected in centers ranging from 3.0 to 22.0%, and was strongly correlated with that of the respective geographic areas. The lack of difference in risk between HCWs who worked in designated COVID-19 departments and those who worked in other departments was reassuring as it indicates that working in high-risk environment did not entail a higher risk of infection, probably because of increased awareness and proper use of PPEs by the employees (1). Monitoring HCWs, both symptomatic and asymptomatic, through screening programs is crucial to rapidly identify and isolate infected subjects and, consequently, to avoid hospital infection outbreaks and to allow healthcare workers to return to work promptly (2). Moreover, alongside screening programs, the implementation of preventive measures and protocols in hospital settings has been shown to be highly effective in reducing the number of cases (3–7). In order to prevent transmission of the virus and protect their parents or children from the pandemic, some HCW have temporarily quarantined themselves from their family. Consistent person-to-person transmission of this novel coronavirus in family settings has been documented (8). Mostly during the first months of the pandemic the quarantine hotels provided the much-needed private space to rest or self-isolate, making life a little less stressful for those battling COVID-19 (Teng et al.) (9). As the COVID-19 pandemic continues, the need to understand and respond to long COVID is increasingly pressing. The term “long COVID” is commonly used to describe signs and symptoms that continue or develop after acute COVID-19. Symptoms such as persistent fatigue, breathlessness, brain fog, and depression could debilitate many millions of people globally. Working on predictive risk factors, such as overweight and obesity, will be a key factor in reducing the incidence of long COVID (10).

In conclusion, this Research Topic broadly encompasses a variety of occupational health-related issues that impact not only workers worldwide, but also and the communities in which they live, highlighting the progress made in the past decade as well as existing and future challenges still needing to be addressed.

## Author contributions

LV has made a substantial, direct, and intellectual contribution to the work and approved it for publication.

## Conflict of interest

The author declares that the research was conducted in the absence of any commercial or financial relationships that could be construed as a potential conflict of interest.

## Publisher's note

All claims expressed in this article are solely those of the authors and do not necessarily represent those of their affiliated organizations, or those of the publisher, the editors and the reviewers. Any product that may be evaluated in this article, or claim that may be made by its manufacturer, is not guaranteed or endorsed by the publisher.
